# Prevalence of Asthma Symptoms and Environmental Triggers Among School-Aged Children in the Eastern Province of Saudi Arabia

**DOI:** 10.7759/cureus.82893

**Published:** 2025-04-24

**Authors:** Masooma J Yaqoob, Hadi A Aqroof, Mohamed J Busehail, Noor M Abdali, Hasan A Alkhayat, Yusuf H Alasfoor, Sayed Mohamed E Alawi, Mark M Abdelsaid, Mahmood A Abdulla, Noor H Alshamlan, Habib A Husain

**Affiliations:** 1 General Practice, Primary Healthcare Centers, Manama, BHR; 2 General Practice, Eastern Health Cluster, Dammam, SAU; 3 College of Medicine, RAK Medical & Health Sciences University, Ras Al-Khaimah, ARE; 4 Pulmonology, King Fahad University Hospital, Al-Khobar, SAU

**Keywords:** asthma, environmental triggers, family history, household smoking, incense use, indoor air pollution, pediatric asthma, prevalence, saudi arabia, school-aged children

## Abstract

Background: Asthma is a prevalent chronic respiratory condition in children, particularly in the Middle East, where environmental and genetic factors contribute to its increasing burden. Indoor environmental exposures such as tobacco smoke, incense use, and carpeting are potential triggers that have not been thoroughly investigated in Saudi Arabia.

Objectives: This study aims to assess the prevalence of asthma symptoms among school-aged children in the Eastern Province of Saudi Arabia and examine their association with specific environmental exposures and familial predisposition.

Methods: A cross-sectional survey was conducted among children aged 6-14 years using a structured, parent-administered questionnaire. The survey collected data on asthma symptoms, physician diagnoses, household smoking, incense use, bedroom carpeting, air conditioning type, and family history of asthma. Chi-square tests and multivariate logistic regression were used to identify associations and independent predictors of wheezing in the past 12 months.

Results: The prevalence of wheezing in the past 12 months was 21.5% (n = 212), and 18.6% (n = 183) of children had a physician diagnosis of asthma. Environmental exposures significantly associated with wheezing included indoor smoking (54.2% vs. 31.3%, p < 0.001; odds ratio (OR): 2.34; 95% confidence interval (CI): 1.65-3.32), daily incense use (48.6% vs. 25.2%, p < 0.001; OR: 2.10; 95% CI: 1.44-3.05), and carpeting in the bedroom (69.3% vs. 48.2%, p < 0.001; OR: 1.83; 95% CI: 1.32-2.55). A family history of asthma also showed a strong association (59.4% vs. 27.5%, p < 0.001; OR: 2.91; 95% CI: 2.05-4.13). Central air conditioning was marginally associated with wheezing (65.1% vs. 57.1%, p = 0.042; OR: 1.41; 95% CI: 1.01-1.96).

Conclusion: This study demonstrates a notable prevalence of asthma symptoms among children in the Eastern Province of Saudi Arabia and identifies key environmental and familial risk factors. Household smoking, incense exposure, and carpeted bedrooms significantly increase the likelihood of asthma symptoms. These findings highlight the need for targeted public health interventions and environmental modifications to mitigate asthma risk in vulnerable pediatric populations.

## Introduction

Asthma is a chronic respiratory condition characterized by airway inflammation and hyper-responsiveness, leading to symptoms such as wheezing, coughing, shortness of breath, and chest tightness [1,2]. It is one of the most common chronic diseases in children worldwide and has become a significant public health issue, with a rising prevalence observed in many parts of the world, including the Middle East [1,3].

Asthma prevalence in children has shown considerable variation across regions and populations, largely due to differences in environmental, genetic, and socio-economic factors. In the Middle East, asthma rates have been reported to be higher than global averages, with some countries experiencing alarming increases in the number of cases [1-5]. Environmental factors play a crucial role in the onset and exacerbation of asthma symptoms in children. Exposure to various environmental allergens and irritants, such as tobacco smoke, air pollution, indoor allergens (e.g., dust mites, pet dander), and household chemicals, has been shown to increase the risk of asthma development and exacerbations. In particular, children living in urban areas are at a higher risk of respiratory conditions due to greater exposure to air pollution, traffic-related pollution, and other environmental hazards [2,6].

Indoor air pollution is a significant contributor to asthma symptoms, particularly in developing regions where the use of tobacco, incense, and inefficient cooking methods is more common. Household smoking has long been recognized as one of the most potent environmental risk factors for childhood asthma, as second-hand smoke can irritate the respiratory tract and trigger asthma attacks in susceptible individuals [4-8]. Studies have demonstrated a clear link between parental smoking and increased rates of asthma-related symptoms in children. Moreover, the use of incense, which is common in many Middle Eastern homes, has been associated with increased respiratory problems, as incense smoke contains particulate matter and other harmful compounds that can trigger asthma attacks [4,9].

Other indoor environmental factors, such as the presence of carpeting in children's bedrooms, have also been identified as risk factors for asthma. Carpets tend to harbor dust mites and other allergens that can exacerbate asthma symptoms in sensitive children. The use of air conditioning is another factor that has been linked to respiratory health, with some studies suggesting that poor maintenance of air conditioning systems may lead to mold growth, which could trigger asthma attacks in vulnerable children [5-11].

In addition to environmental triggers, genetic factors play an important role in the development of asthma. A family history of asthma significantly increases the likelihood of a child developing the condition. It is well-established that asthma has a strong genetic component, and children with one or both parents suffering from asthma are at a higher risk of experiencing asthma symptoms themselves. This interaction between genetic predisposition and environmental exposures is critical in understanding asthma’s multifactorial nature and its increasing prevalence in children [10,12].

While many studies have explored the prevalence and environmental triggers of asthma in other parts of the world, research on asthma in Saudi Arabia remains limited. Most studies on asthma in the country have focused on specific cities or regions, often without considering the wide range of environmental factors that may contribute to asthma symptoms [5-11]. As urbanization increases in Saudi Arabia, there is an urgent need for studies that investigate how local environmental factors, such as smoking, incense use, air conditioning, and indoor allergens, interact with genetic factors to influence asthma prevalence in school-aged children [11,12].

This study aims to fill this gap by providing a comprehensive assessment of asthma symptoms and their associations with environmental triggers in the Eastern Province of Saudi Arabia. The findings of this study will contribute to a better understanding of the environmental determinants of asthma in this region and will inform public health strategies aimed at reducing asthma symptoms and improving the quality of life for affected children.

## Materials and methods

Study design

This study was a cross-sectional survey conducted to assess the prevalence of asthma symptoms and associated environmental triggers in school-aged children in the Eastern Province of Saudi Arabia. The study was designed to identify key environmental exposures related to asthma symptoms, including wheezing, and to determine factors that may influence the development or exacerbation of asthma-like symptoms in children.

Study population

The study sample consisted of school-aged children aged 6-14 years who were enrolled in schools in the Eastern Province of Saudi Arabia. The children were selected through a stratified random sampling technique, which ensured the inclusion of both urban and rural residents, as well as children attending both public and private schools.

The inclusion criteria for this study were as follows: children aged 6-14 years, enrolled in either public or private schools in the Eastern Province, and whose parents or guardians provided informed consent for participation. The exclusion criteria included children with a history of chronic respiratory conditions other than asthma, such as cystic fibrosis, as well as children whose parents did not consent to participation in the study.

Data collection

Data were collected using a structured, self-administered questionnaire distributed to parents or guardians of participating children (Appendix A). The questionnaire was designed to assess demographic characteristics, asthma symptoms, and environmental exposures that may contribute to wheezing and asthma-like symptoms. The questionnaire was validated in a pilot study conducted prior to the main data collection to ensure clarity, reliability, and appropriateness for the target population.

The survey included the following main sections: The first section gathered demographic information, including age, sex, school type, and residential area. The second section focused on asthma symptoms, asking about wheezing in the past 12 months, physician-diagnosed asthma, and family history of asthma. The third section addressed environmental exposures, with questions regarding household smoking, incense use, the presence of a carpet in the child's bedroom, and the type of air conditioning in the home.

All questionnaires were filled out in the presence of a trained data collector to ensure accuracy and completeness. The study was approved by the ethical review board of the institution, and written informed consent was obtained from the parents or guardians of all participants.

Exposure and outcome variables

The primary outcome variable of this study was the presence of asthma symptoms, specifically wheezing, in the past 12 months. The secondary outcome was physician-diagnosed asthma, as reported by the parents. Several environmental factors were considered as potential explanatory variables for asthma symptoms. These included smoking in the household, categorized as "Yes (inside)," "Yes (outside only)," or "No"; incense use, categorized as "Daily," "Occasionally," "Rarely," or "Never"; the presence of carpet in the bedroom, categorized as "Yes" or "No"; air conditioning type, categorized as "Central," "Split unit," "Window," or "None"; and family history of asthma, categorized as "Yes" or "No."

Statistical analysis

Data were analyzed using the IBM SPSS Statistics for Windows, Version 25 (Released 2017; IBM Corp., Armonk, New York, United States). Descriptive statistics were used to summarize demographic characteristics, asthma symptoms, and environmental exposures. Continuous variables were expressed as mean ± standard deviation (SD), and categorical variables were presented as frequencies and percentages.

Chi-square tests were used to examine the association between asthma symptoms (wheezing and physician-diagnosed asthma) and various environmental exposures (household smoking, incense use, carpet in the bedroom, air conditioning type, and family history of asthma). Statistical significance was set at p < 0.05.

To identify independent predictors of wheezing in the past 12 months, a multivariate logistic regression analysis was performed. The model included environmental variables found to be significantly associated with wheezing in univariate analyses. Odds ratios (OR) with 95% confidence intervals (CI) were calculated to assess the strength of these associations. Variables such as age, sex, and residence were included as potential confounders in the model.

## Results

Participant characteristics

A total of 984 children participated in the study. The mean age of the children was 9.6 years (SD ±2.3), with the largest proportion of participants aged 9-11 years (37.7%). Males made up 51.4% of the sample, while females represented 48.6%. Most of the children attended public schools (70.4%) and lived in urban areas (71.9%), with fewer participants residing in rural (17.9%) or suburban (10.3%) areas (Table 1).

**Table 1:** Demographic characteristics of the study population (n = 984) Frequencies and percentages are provided for each category.

Variable	Category	n (%)
Age group (years)	6-8	312 (31.7)
	9-11	371 (37.7)
	12-14	301 (30.6)
Sex	Male	506 (51.4)
	Female	478 (48.6)
School type	Public	693 (70.4)
	Private	291 (29.6)
Residence	Urban	707 (71.9)
	Rural	176 (17.9)
	Suburban	101 (10.3)

Prevalence of asthma symptoms

Regarding asthma symptoms, 212 children (21.5%) reported wheezing in the past 12 months, while 772 children (78.5%) did not experience wheezing during the same period. A physician-diagnosed asthma condition was reported by 183 parents (18.6%) of the children, whereas 801 children (81.4%) had no history of an asthma diagnosis. These findings suggest a moderate prevalence of asthma symptoms among school-aged children in this population (Table 2).

**Table 2:** Prevalence of asthma symptoms and environmental exposures (n = 984) The frequencies and percentages of each category are reported.

Variable	Category	n (%)
Wheezing in the past 12 months	Yes	212 (21.5)
	No	772 (78.5)
Physician-diagnosed asthma	Yes	183 (18.6)
	No	801 (81.4)
Smoking in household	Yes (inside)	136 (13.8)
	Yes (outside only)	221 (22.5)
	No	627 (63.7)
Use of incense at home	Daily	298 (30.3)
	Occasionally	419 (42.6)
	Rarely	154 (15.7)
	Never	113 (11.5)
AC type at home	Central	579 (58.8)
	Split unit	288 (29.3)
	Window	97 (9.9)
	None	20 (2.0)
Carpet in child's bedroom	Yes	519 (52.7)
	No	465 (47.3)

Environmental exposures such as smoking, incense use, air conditioning, and carpeting in the bedroom were explored as potential triggers for wheezing. The study found that 13.8% (n = 136) of households had indoor smoking, 22.5% (n = 221) had smoking outside, and 63.7% (n = 627) reported no smoking at all. Daily exposure to incense was reported by 30.3% (n = 298) of the participants, while 42.6% (n = 419) had occasional incense use. Furthermore, 52.7% (n = 519) of children had carpeting in their bedrooms, while 47.3% (n = 465) did not. In terms of air conditioning, 58.8% (n = 579) of households used central air conditioning, 29.3% (n = 288) used split units, 9.9% (n = 97) used window units, and 2.0% (n = 20) of participants reported no air conditioning in their homes.

Associations between environmental triggers and wheezing

Environmental factors were significantly associated with wheezing in the past 12 months. A chi-square test revealed that smoking in the household (54.2% vs. 31.3%, p < 0.001), daily incense use (48.6% vs. 25.2%, p < 0.001), and carpeting in the child’s bedroom (69.3% vs. 48.2%, p < 0.001) were all strongly associated with wheezing. Central air conditioning was marginally associated with wheezing (65.1% vs. 57.1%, p = 0.042). Moreover, a family history of asthma was also a significant factor, with a higher proportion of wheezing children having a family history of asthma compared to those without (59.4% vs. 27.5%, p < 0.001) (Table 3).

**Table 3:** Association between environmental triggers and the occurrence of wheezing in the past 12 months (n = 984) The chi-square (χ²) test was used. Statistical significance was considered at p < 0.05. Test statistic (χ²) values are shown.

Variable	Wheezing (n = 212), n (%)	No Wheezing (n = 772), n (%)	χ² value	p-value
Smoking in household	115 (54.2)	242 (31.3)	37.19	<0.001
Daily incense use	103 (48.6)	195 (25.2)	39.77	<0.001
Carpet in bedroom	147 (69.3)	372 (48.2)	27.40	<0.001
Central AC	138 (65.1)	441 (57.1)	4.12	0.042
Family history of asthma	126 (59.4)	212 (27.5)	65.21	<0.001

Multivariate logistic regression analysis

Multivariate logistic regression analysis was conducted to identify independent predictors of wheezing. After adjusting for confounders, the following factors were found to be significant predictors of wheezing in the past 12 months: smoking in the household (adjusted OR 2.34; 95% CI, 1.65-3.32; p < 0.001), daily incense use (OR 2.10; 95% CI, 1.44-3.05; p < 0.001), carpeting in the bedroom (OR 1.83; 95% CI, 1.32-2.55; p < 0.001), and family history of asthma (OR 2.91; 95% CI, 2.05-4.13; p < 0.001). Central air conditioning was also marginally associated with wheezing (OR 1.41; 95% CI, 1.01-1.96; p = 0.045), while age, sex, and urban residence were not significant predictors of wheezing (Table 4).

**Table 4:** Results of multivariate logistic regression analysis examining the predictors of wheezing in the past 12 months The table includes adjusted odds ratios (OR) and 95% confidence intervals (CI) for each predictor, as well as p-values to indicate statistical significance.

Predictor Variable	Adjusted OR (95% CI)	p-value
Smoking in household	2.34 (1.65-3.32)	<0.001
Daily incense use	2.10 (1.44-3.05)	<0.001
Carpet in bedroom	1.83 (1.32-2.55)	<0.001
Central AC	1.41 (1.01-1.96)	0.045
Family history of asthma	2.91 (2.05-4.13)	<0.001
Age (per year increase)	0.95 (0.89-1.01)	0.09
Male sex	1.12 (0.83-1.50)	0.44
Urban residence	1.06 (0.73-1.54)	0.75

## Discussion

This study investigated the prevalence of asthma symptoms and their association with environmental triggers in school-aged children in Saudi Arabia. The results show that wheezing in the past 12 months was reported by 21.5% of children, and 18.6% had been diagnosed with asthma by a healthcare provider. We identified several significant environmental factors associated with wheezing, including household smoking, daily incense use, carpeted bedrooms, and a family history of asthma. Additionally, multivariate analysis revealed that these factors were independently associated with increased odds of wheezing, underscoring the potential role of environmental exposures in the pathogenesis of asthma symptoms in children.

The reported prevalence of wheezing in the past 12 months (21.5%) aligns with similar studies in the Middle East, where asthma and allergic diseases have emerged as significant public health concerns among children. For example, a study in Qatar reported a similar prevalence of 21.3% for wheezing in the past year among school-aged children, suggesting that asthma symptoms are highly prevalent in the region. The rate of physician-diagnosed asthma (18.6%) is also consistent with other regional studies, indicating that asthma diagnoses in Saudi Arabia are relatively in line with international patterns. These findings reinforce the importance of monitoring and managing asthma symptoms in children, especially in urban areas where environmental exposures may be more concentrated [10-14].

One of the most striking findings of this study was the strong association between household smoking and wheezing in children. Children exposed to indoor smoking were more than twice as likely to report wheezing compared to those not exposed. These findings are consistent with extensive literature that demonstrates the harmful effects of tobacco smoke on children’s respiratory health [7,9,11]. Passive smoke exposure has been well-established as a major risk factor for asthma symptoms and exacerbations in children. Smoking in the household, whether indoors or outdoors, was significantly associated with wheezing, which highlights the need for interventions targeting smoking prevention and cessation in households to reduce asthma-related morbidity in children.

Exposure to incense smoke was another major environmental factor linked to wheezing in this study. Daily incense use was significantly more common among children with wheezing (48.6%) than those without (25.2%). Incense use has been associated with respiratory symptoms and asthma exacerbations due to the release of particulate matter and other irritants. In many parts of the Middle East, incense is commonly used for cultural and religious purposes, which may inadvertently contribute to indoor air pollution. The high prevalence of daily incense exposure in this study underscores the need for awareness campaigns about the potential health risks of indoor air pollution and the promotion of alternatives to incense use in households with children at risk for asthma [15,16].

The presence of carpeting in the child's bedroom was also found to be strongly associated with wheezing. Children living in homes with carpeted bedrooms were significantly more likely to report wheezing compared to those without carpeting. Carpets are known to trap dust, allergens, and other particulate matter, which may exacerbate respiratory symptoms, particularly in children with asthma or other allergic conditions [5,7,13]. The association between carpeting and wheezing is well-supported by previous studies that have found that carpeted homes tend to have higher levels of allergens, including dust mites and pet dander, which are known triggers for asthma exacerbations. This finding highlights the importance of creating asthma-friendly home environments, particularly by reducing allergen exposure through interventions such as regular cleaning and minimizing the use of carpets in children’s bedrooms.

The strong association between a family history of asthma and wheezing in this study is consistent with the well-established genetic component of asthma. A family history of asthma has long been recognized as a key risk factor for the development of asthma in children. This study reinforces the importance of considering family history when assessing the risk of asthma in children and highlights the need for early identification and management of at-risk children. Families with a history of asthma may benefit from educational interventions aimed at reducing environmental triggers and promoting early asthma care [9-11].

Interestingly, the use of central air conditioning was also found to be marginally associated with wheezing. Although this association was not as strong as the other environmental factors, it warrants further investigation. Previous studies have suggested that air conditioning use may help reduce exposure to outdoor allergens by filtering air, yet it can also lead to indoor air quality issues such as mold growth or inadequate ventilation. In this study, central air conditioning was slightly more common among children who reported wheezing (65.1%) compared to those who did not (57.1%). Further research is needed to better understand the complex relationship between indoor air conditioning, air quality, and asthma symptoms in children.

Strengths and limitations

This study has several strengths, including its relatively large sample size (984 children) and the use of standardized survey instruments to assess asthma symptoms and environmental exposures. The study's cross-sectional design provides a snapshot of the prevalence of asthma symptoms and associated environmental risk factors in Saudi school-aged children.

However, there are also several limitations to consider. As a cross-sectional study, we cannot infer causality from the observed associations. The reliance on self-reported data may have introduced recall bias, particularly regarding environmental exposures like smoking and incense use. Additionally, while we controlled for several potential confounders, there may be other unmeasured factors, such as diet or socioeconomic status, that could influence asthma symptoms. Finally, the study was conducted in a specific region of Saudi Arabia (Eastern Province), which may limit the generalizability of the findings to other parts of the country or the broader Middle East.

## Conclusions

This study highlights the significant role of environmental factors in the prevalence and exacerbation of asthma symptoms in school-aged children in the Eastern Province of Saudi Arabia. Our findings underscore the impact of household smoking, incense use, and the presence of carpets in children's bedrooms as key environmental triggers for asthma symptoms. The strong association between these factors and asthma, along with the increased risk linked to family history, calls for targeted public health interventions to reduce exposure to these environmental risks. Furthermore, this research contributes valuable data to the growing body of knowledge on pediatric asthma in the Middle East, providing evidence to inform preventive strategies, healthcare policies, and asthma management practices aimed at improving the health and well-being of children in this region. Continued research and interventions are necessary to further elucidate the complex interplay between genetic predisposition and environmental exposures in asthma development and management.

## Appendices

Appendix A

Section 1: Demographic Information

Child's age: ___ years

Child's sex: ☐ Male ☐ Female

School type: ☐ Public ☐ Private

Residential area: ☐ Urban ☐ Suburban ☐ Rural

Section 2: Asthma Symptoms

Has your child experienced wheezing or whistling in the chest in the past 12 months? ☐ Yes ☐ No

Has your child ever been diagnosed with asthma by a physician? ☐ Yes ☐ No

Does anyone in your immediate family (parents, siblings) have a history of asthma? ☐ Yes ☐ No

Section 3: Environmental Exposures

Is there smoking in your household? ☐ Yes, inside the home ☐ Yes, outside only ☐ No

How often is incense burned in your home? ☐ Daily ☐ Occasionally ☐ Rarely ☐ Never

Does your child have a carpet or rug in their bedroom? ☐ Yes ☐ No

What type of air conditioning is used in your home? ☐ Central ☐ Split unit ☐ Window ☐ None
